# Draft Genome Sequence of *Burkholderia puraquae* Type Strain CAMPA 1040, Isolated from Hospital Settings in Córdoba, Argentina

**DOI:** 10.1128/genomeA.01302-17

**Published:** 2017-11-22

**Authors:** Mariana Leguizamon, Walter O. Draghi, Patricia Montanaro, Andy Schneider, Claudia I. Prieto, Pablo Martina, Antonio Lagares, Peter Lasch, Alejandra Bosch

**Affiliations:** a CINDEFI, CCT-CONICET–La Plata, Facultad de Ciencias Exactas, Universidad Nacional de La Plata, La Plata, Buenos Aires, Argentina; b IBBM (Instituto de Biotecnología y Biología Molecular), CCT-CONICET–La Plata, Facultad de Ciencias Exactas, Universidad Nacional de La Plata, La Plata, Buenos Aires, Argentina; c Hospital Santísima Trinidad de Córdoba, Córdoba, Argentina; d Proteomics and Spectroscopy Unit (ZBS6) at the Centre for Biological Threats and Special Pathogens, Robert Koch-Institut, Berlin, Germany

## Abstract

We report here the draft genome sequence of *Burkholderia puraquae* type strain CAMPA 1040, a member of the *Burkholderia cepacia* complex. This strain, isolated from a hemodialysis water reservoir, harbors several stress tolerance genes, such as the systems for low oxygen survival, for copper tolerance, and for osmotic stress resistance.

## GENOME ANNOUNCEMENT

*Burkholderia puraquae* is a recently described member of the *Burkholderia cepacia* complex (Bcc), a group of at least 22 Gram-negative related bacterial species (1–7). Although Bcc bacteria are mainly recognized as opportunistic pathogens of cystic fibrosis or immunocompromised patients (8, 9), the most diverse natural, industrial, and environmental niches (such as agricultural soils, plant root nodules, industrial products, dialysis water, and medical instruments, among others), can be colonized and infected by these species, indicating the wide versatility of this bacterial complex (10–12).

The isolates assigned to *Burkholderia puraquae* until now have been recovered from different environmental niches, such as hemodialysis water reservoirs located at two different hospitals and from agricultural soil (7). In order to obtain a better understanding of this species, whole-genome shotgun sequencing of *Burkholderia puraquae* type strain CAMPA 1040 (=LMG 29660^T^ =DSM 130137^T^), isolated from a hemodialysis water reservoir at the Hospital Santísima Trinidad in Córdoba city, Argentina, in 2011, was performed. Colonies of a fresh culture grown on LB agar plates were selected. Genomic DNA was extracted using the QIAamp DNA minikit (Qiagen, Hilden, Germany), and the sequencing was done in 300-nucleotide (nt) paired-end mode on an Illumina MiSeq version 3 sequencing platform at LGC Genomics (Berlin, Germany). A total of 4,073,044 reads were obtained, which were trimmed and assembled *de novo* using the A5 pipeline (13). Eighty-two scaffolds were obtained (longest scaffold, 982,812 bp; *N*_50_, 290,133 bp), with 85× coverage. The total size of the assembly was 8,098,134 bp, with 66.5% GC content. Scaffolds were submitted to GenBank for gene annotation, which was implemented using the NCBI Prokaryotic Genome Annotation Pipeline (PGAP) (14); 7,182 coding genes, 60 tRNAs, 11 rRNAs, and 75 ribosomal proteins were found.

Analysis of the automatically annotated scaffolds showed the presence of several systems acting on defense of bacterial stress. Among them, the homolog of the gene cluster designated the low-oxygen-activated (*lxa*) locus was present in this strain (15). This locus is a coregulated 50-gene cluster significantly upregulated during growth under low oxygen conditions in *Burkholderia cenocepacia* strain J2315. The gene cluster is involved in a number of cellular functions, such as metabolism, carbohydrate transport, electron transfer, and regulation of stress-related proteins (15). In addition, several systems involved in copper homeostasis were present in the genome as CopSR and CusSR (two-component regulatory systems), involved in sensing and inducing the expression of Cu resistance determinants to deal with periplasmic copper excess, and the ScsADCB locus, which contributes to cellular copper tolerance (16). Furthermore, several genes involved in osmotic stress defense through the synthesis of glycine betaine from choline were present in the genome. The overall analysis showed several systems concerning cell defense against biotic and abiotic stress, which in part could explain the adaptability of this bacterial species to diverse environments.

### Accession number(s).

The *Burkholderia puraquae* CAMPA 1040^T^ whole-genome shotgun (WGS) project has been submitted to DDBJ/EMBL/GenBank under the accession number NBYX00000000. This version of the project presented here is NBYX01000000.
